# Mitochondrial defects potentiate seizure development in Wolf-Hirschhorn syndrome

**Published:** 2014-05

Wolf-Hirschhorn syndrome (WHS) is a gene-deletion disorder characterised by growth complications, craniofacial abnormalities, intellectual disabilities and epileptic seizures. The deletion of genes within two critical chromosomal regions is responsible for many of these features but, because these deletions span multiple genes, defining the phenotypic contribution of a single gene is challenging. Haploinsufficiency of *LETM1*, which encodes a mitochondrial antiporter protein, has been linked to seizure development and other features typical to WHS, suggesting that mitochondrial dysfunction could contribute to pathogenesis. To explore this possibility, Mark O’Driscoll and colleagues investigated mitochondrial function in WHS-patient-derived cell lines. The authors confirm that there is a reduction in *LETM1* expression in cells from individuals with WHS, and also demonstrate that LETM1 deficiency segregates with seizure development in the patient cohort. Furthermore, they show that mitochondria in LETM1-deficient cells have altered calcium levels, and that these mitochondria display dysfunctional voltage-gated channels and atypical membrane potentials. Using mouse and human cell lines, the group reveals that a decrease in *LETM1* expression alone is sufficient to generate abnormal mitochondrial phenotypes. Collectively, these findings lend weight to the hypothesis that mitochondrial dysfunction as a result of *LETM1* haploinsufficiency underlies seizure development (and potentially other clinical features) in WHS. This work relates haploinsufficiency of a single gene to a key phenotype associated with the disorder, and suggests the potential therapeutic benefit of correcting mitochondrial defects. **Page 535**

This summary was written by Anna J. Moyer, who is currently in the third year of a dual Bachelor’s/Master’s programme in Biology at the University of Alabama.

